# Strategy Use on Digital Cognitive Assessment Differs Between Individuals with and without Elevated Phosphorylated Tau 217 in Cognitively Normal Older Adults: Results from the BioFINDER‐Brown Study

**DOI:** 10.1002/alz.095272

**Published:** 2025-01-09

**Authors:** Alyssa N. De Vito, Hannah E Joyce, Molly Lawrence, Louisa I. Thompson, Phillip Caffery, John P.K. Bernstein, Steve Salloway

**Affiliations:** ^1^ Butler Hospital Memory and Aging Program, Providence, RI USA; ^2^ Alpert Medical School of Brown University, Providence, RI USA; ^3^ Butler Hospital, Providence, RI USA; ^4^ Cambridge Cognition, Cambridge, Bottisham United Kingdom; ^5^ Brown University, Providence, RI USA

## Abstract

**Background:**

New immunotherapies for Alzheimer’s Disease (AD) have shown promise in slowing disease progression and are most effective in the early stages of the disease. New, low‐cost methodologies are needed to detect early AD pathological change. Digital cognitive tools may represent a cost effective, efficient, and sensitive method to detect early AD‐related pathological change. The present analysis utilizes data from the BioFINDER‐Brown study to investigate whether individuals with and without elevated plasma phosphorylated tau‐ 217 (p‐tau_217_) demonstrate differential performance on digital cognitive assessments of spatial working memory (SWM) and paired associates learning (PAL).

**Method:**

90 cognitively normal (CN; *M*_MMSE_ = 29.00) older adults (*M*_age_ = 63.36, 72.2% female, 95.6% white, 5.6% Latinx) completed a blood draw to evaluate plasma p‐tau_217_ and two brief tablet‐based digital cognitive assessments: SWM and PAL. as part of screening for the BioFINDER‐Brown study. Approximately 42% of the sample (n = 38) had elevated plasma p‐tau_217_. Independent samples t‐tests were used to determine whether performance on these measures differed between individuals with and without elevated p‐tau_217_. Lower scores on both measures are indicative of better performance.

**Result:**

The total number of errors on the SWM was not found to differ between individuals with and without elevated plasma p‐tau_217_ (*t*(89) = ‐1.33, *p* = 0.093). However, differences in strategy use were found on the SWM task (*t*(89) = ‐2.11, *p* = 0.019); Individuals with elevated p‐tau_217_ had a less efficient strategy and required more trials to complete the task (M = 8.79 trials) than non‐elevated individuals (M = 7.65 trials). Group differences between those with and without elevated p‐tau_217_ on the PAL task approached significance (*t*(89) = 1.08, *p* = .075), with worse performance in those with elevated p‐tau_217_.

**Conclusion:**

The preliminary findings suggest that group differences may exist for process scores (i.e., strategy use) but not overall performance on digital cognitive assessments between individuals with and without elevated plasma p‐tau_217_. Future research should evaluate 1) which digital assessments are most sensitive to AD‐related pathological change and 2) whether digital cognitive assessments can be used in conjunction with novel, affordable plasma biomarkers to improve early detection of AD.

